# Mapping Current Evidence and Research Gaps in Nutrition for Healthy Children and Adolescents: A Scoping Review

**DOI:** 10.3390/nu18101578

**Published:** 2026-05-15

**Authors:** Diana Montiel-Ojeda, Lucía Méndez-Sánchez, Desiree Lopez-Gonzalez, Patricia Clark

**Affiliations:** 1 Clinical Epidemiology Research Unit, Children’s Hospital of Mexico Federico Gomez, Faculty of Medicine, National Autonomous University of Mexico UNAM, Mexico City 06720, Mexico; diana-montiel@facmed.unam.mx; 2 Cochrane Mexico UNAM Associated Group, Library and Information System, Faculty of Medicine, National Autonomous University of Mexico UNAM, Mexico City 04510, Mexico; lmendez@facmed.unam.mx

**Keywords:** child, adolescent, dietary patterns, dietary intake, diet, nutrition

## Abstract

**Introduction:** A healthy diet is crucial for healthy growth and development during childhood and adolescence. Although evidence in this field is expanding rapidly, the focus has been on disease-oriented nutrition rather than on nutrition for healthy children and adolescents; thus, we aimed to identify key research gaps to guide future studies and policy development. **Methods**: For this scoping review, we systematically searched systematic reviews, clinical guidelines, and position papers. The literature search covered publications from the last eight years to provide the latest information on pediatric nutrition. A basic critical appraisal was performed to assess the quality of the evidence. **Results**: We included 14 studies. Six major topics were identified as the most frequently reported: healthy dietary patterns, sustainable dietary patterns, macronutrients and micronutrients, sugar intake, beverages and sugary drinks, and dairy products. **Conclusions**: Current information on healthy diets for children and adolescents focuses mainly on dietary patterns. However, regionalized information is lacking, as few health agencies report on specific dietary modifications, especially in developing or transition countries.

## 1. Introduction

Globally, there are approximately 45.4 million children under the age of 5 who are underweight, of whom 13.6 million are severely underweight. Conversely, about 39 million children under 5 are overweight or obese, and at least 80% are expected to suffer obesity throughout childhood, adolescence, and adulthood. These and other diet-related conditions could be prevented and treated early through a healthy diet and lifestyle [1,2,3]. A healthy diet supports good health and helps prevent disease. It ensures sufficient intake of nutrients and beneficial substances from nutritious foods while avoiding harmful substances [4]. A dietary pattern refers to the regular combination of foods and beverages that a person habitually consumes [5].

In recent years, significant advances have been made in childhood and adolescent diet and nutrition in the context of disease. However, this is not the case for children and adolescents in a healthy context; evidence has not advanced at the same pace. When we refer to a healthy context, we mean children and adolescents who do not have diseases or nutritional disorders that affect their health status. This is important because healthy children and adolescents also require preventive health surveillance to prevent long-term health consequences. It is also crucial to take into account that children’s and adolescents’ health involves eating behavior, which is shaped not only by nutrient availability and dietary guidelines but also by psychological, cultural, and social determinants [6,7].

There are different methodological strategies for identifying information gaps; one is scoping reviews, which aim to identify and methodically map existing information on a topic of interest [8]. For this reason, we decided to conduct a scoping review to map and categorize the available evidence on nutrition in healthy children and adolescents and to identify key research gaps to guide future studies and policy development.

## 2. Materials and Methods

### 2.1. Protocol and Registration

The methodology for this scoping review followed the recommendations of Tricco et al. [9] and Cooper et al. [10], as well as the guide for scoping reviews in the JBI Manual for Evidence Synthesis [11]. In addition, for this review, some recommendations from the Cochrane Handbook for Systematic Reviews of Interventions, version 5.1.0 [12], were considered. The protocol for this scoping review was registered on the Open Science Framework (OSF) registry: osf.io/p6evd.

### 2.2. Eligibility Criteria

The literature search, guided by the PEOS framework [11] (Supplementary Table S1), was conducted to answer the following question: What evidence-based recommendations relate to children’s and adolescents’ nutritional health, and what are the current research gaps? In order to find studies that show current evidence and research gaps in nutrition for healthy children and adolescents. For this study, studies that included information on children and adolescents aged 2–19 years with no diagnosed chronic disease, acute illness, or nutritional disorder at the time of assessment were considered.

### 2.3. Search

An initial pilot search was conducted to identify the main nutritional health outcomes of interest in this age group, and the search strategy was adapted for each database using the following MeSH (Medical Subject Headings) terms: Dietary Guidelines, Food-based Dietary Guidelines, Diet, and Children. Following this pilot search, a systematic search of the scientific literature was conducted to identify relevant publications using MeSH search terms, adapting each search engine under the following terms: ‘Child’, ‘Adolescent’, ‘Dietary Guidelines Food-based’, ‘Dietary Guidelines’, ‘Diet’, ‘Vitamins’, ‘Minerals’, ‘Sugar’, ‘Sweeteners’, ‘Vegetarianism’, ‘Plant-based diet’ and ‘Dairy’. All the search terms are listed in Supplementary Table S2. Only articles in English and Spanish were included.

For this scoping review, and as a result of our pilot search, the review focused on the following six outcome categories: (1) healthy dietary patterns, (2) macronutrients and micronutrients, (3) sustainable diets, (4) sugar, (5) beverages and sugary drinks, and (6) dairy products. For macronutrients and micronutrients, only the data available in the publications included in this review were reported, such as fatty acids and vitamin D. Other nutrients were not included because no data on them were reported in the included publications.

### 2.4. Information Sources

According to the scoping review methodology [9,11], several types of studies can be included in this type of review. For our scoping review, we decided to include only systematic reviews (with and without meta-analyses), position papers, and clinical guidelines, as these study types are methodologically more reliable and can cover a wide range of clinical information on nutrition. The studies had to specifically include children and adolescents (aged 2–19 years). The main characteristic of the studies was that they assessed children and adolescents in a healthy context, which was the main purpose of this review. The studies included were from 2016 to 2024. Although the initial registered protocol covered up to 2022, the authors of this scoping review decided to extend the search years to improve the integration of the results, given the information available up to that point. Studies were excluded if they did not meet the inclusion criteria or if the full text of the article was not available in English or Spanish.

The e-libraries used for the research were PubMed (Medline), Cochrane Library, Trip Database, Epistemonikos, the Mexican Social Security Institute’s repository of clinical practice guidelines, the master catalog of the National Center for Health Technology Excellence (CENETEC), and the guidelines of the National Institute for Health and Care Excellence (NICE). In addition, a manual, intentional search of bibliographical references in relevant articles was conducted. The search was carried out between March and May 2025.

### 2.5. Selection of Sources of Evidence

The process was iterative and included a literature search, refinement of the search strategy, and a complete review of the articles for inclusion. Two authors (DMO and PC) searched for eligible articles. The six outcome categories were established a priori as part of the registered protocol, guided by the pilot search. An initial screening of articles was performed, excluding those whose titles and/or abstracts did not meet the inclusion criteria or the expected outcomes. Both authors performed separate full-text exclusions of the studies. Any discrepancies in the inclusion or exclusion of studies were resolved by a third author (LMS).

### 2.6. Data Extraction and Synthesis

Two reviewers (DMO and PC) extracted all the data using specific formats for this review (compiled by the authors in Excel spreadsheets for MS).

### 2.7. Critical Appraisal of Sources of Evidence

Two authors (DMO and PC) independently analyzed the quality appraisal of the included studies. For systematic reviews, the information was assessed using the AMSTAR 2 tool [13]. For the position papers, the Evidence Analysis Manual for Review Studies from the Academy of Nutrition and Dietetics [14] was used as an assessment instrument. Finally, the RIGHT tool [15] was used to assess clinical guidelines. Owing to the scoping review methodology and the heterogeneity of the information, a meta-analysis of the main outcomes was not possible.

## 3. Results

### 3.1. Selection of Sources of Evidence

**Figure 1:**
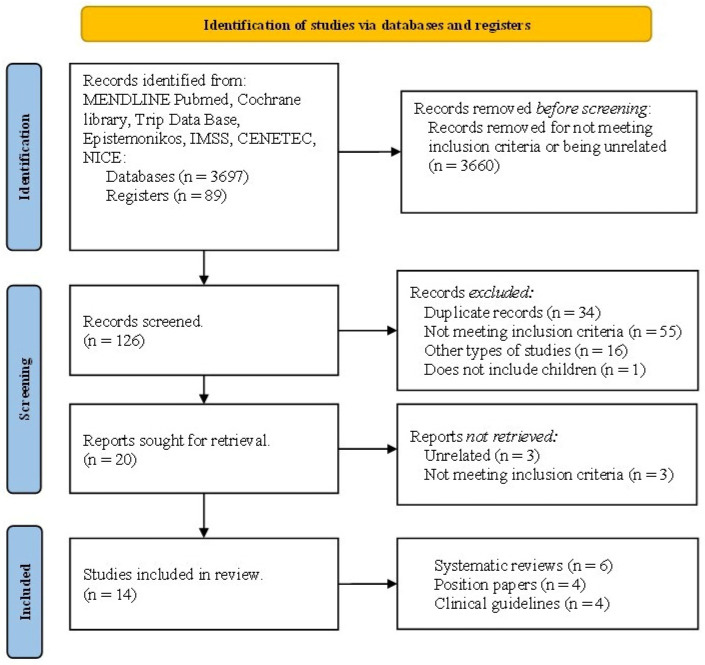
PRISMA flow diagram.

Out of 3786 titles identified, 126 abstracts were screened, and 20 studies were assessed in full for eligibility (PubMed = 13, Trip Database = 5, GPC IMSS = 2). Of these, only 14 articles met the inclusion criteria; six were excluded (see Supplementary Table S3). The kappa value after pairwise selection was 0.606 (*p* < 0.0001), indicating substantial agreement among the evaluators. The disagreements were resolved by consensus, with a third reviewer (LMS). Among the 14 studies selected, six are systematic reviews, four are position papers, and four are clinical guidelines (Figure 1).

Four [16,17,18,19] of the six systematic reviews reported an approximate sample size of 343,908 children and adolescents. Two reviews analyzed clinical trials [16,17], and the remaining four [18,19,20,21] included mixed information from clinical trials and other observational studies. All position papers [22,23,24,25] covered the pediatric population, but only one included children aged 0–5 years. Finally, only one of the clinical guidelines [26] focused on the pediatric population, whereas the remaining three [27,28,29] were aimed at the general population but provided some information on children and adolescents.

### 3.2. Content and Characteristics of the Evidence on Nutrition in Healthy Children and Adolescents

For the six outcomes of this scoping review, five studies focused on healthy dietary patterns [20,23,26,27,28], two studies on sustainable dietary patterns [19,29], two studies on dairy products [16,18], and one study on each of the remaining outcomes: macronutrients—fatty acids (SFAs) [17], micronutrients—vitamin D [21], sugar [22] and beverages—sugary drinks [24,25] (Table 1).

In what follows, the results from each included study are described according to the six outcomes used in this scoping review.

#### 3.2.1. Dietary Patterns

Two studies specifically addressed children’s and adolescents’ healthy dietary patterns [20,28]. In two studies, promoting a Mediterranean diet (MD) pattern was associated with beneficial health effects, including the prevention of overweight and obesity in childhood. In contrast, an unhealthy dietary pattern, or ‘Western or modern’ pattern, was associated with a higher body mass index (BMI), increasing the risk of being overweight or obese in childhood and adolescence. Despite this, a systematic review reported that, after assessing adherence to this diet using different instruments, adherence in the pediatric population is low [20].

One strategy proposed to eradicate harmful dietary patterns is nutritional education. According to a position paper [23] and a Mexican clinical guideline [27], nutritional education is a key factor in children’s and adolescents’ health and aims to strengthen and maintain healthy eating habits.

#### 3.2.2. Sustainable Dietary Patterns

Two studies [19,29] reported recommendations for healthy and sustainable diets for children and adolescents. The first study was a systematic review [19]. In this study, a dietary pattern is defined as sustainable when it is nutritionally appropriate; has a low environmental impact; contributes to the protection of biodiversity and the ecosystem; and is culturally acceptable, safe, accessible, and affordable. This study also adopts the MD pattern for children and adolescents and reports that it has the strongest association with sustainability and has been evaluated most in the pediatric population.

The second study was a compendium of clinical guidelines of the WHO [29], and in this study, the authors recommend that children and adolescents should follow a sustainable diet pattern that promotes well-being and reduces environmental impact, based on a diet high in whole grains and a variety of fruits and vegetables, nuts, and seeds and low in foods of animal origin and discretionary foods (such as sugary drinks). The study also recommends promoting and educating healthy eating and sustainability from an early age to maximize benefits and help maintain this diet pattern from childhood to adulthood.

#### 3.2.3. Macronutrients and Micronutrients

According to a systematic review [20], no reliable evidence has been reported on any dietary pattern based on macronutrient distribution associated with growth, body composition, or the risk of overweight or obesity. Because of the heterogeneity of the evidence, the authors reported that it was not possible to rate the quality of the evidence with any degree of confidence.

##### Fatty Acids

A systematic review [17] found that after the implementation of nutritional counseling for children, parents, and primary caregivers, participants exhibited a clinically significant decrease in total cholesterol. The studies included in this paper reported a minimum follow-up of 4 months and a maximum of 19 years. The recommended SFA intake ranged from 7 to 13% of total daily energy intake. The study concluded that cholesterol levels could be maintained within normal ranges when SFA intake was kept below 10% of total energy intake (TEI).

##### Vitamins (Vitamin D)

For vitamin D, one systematic review focused on adolescents [21] concluded that current intake recommendations range from 200 to 1000 IU/day, depending on the context in which the population lives and some other considerations, such as skin color, geographical area of residence, or health conditions of adolescents. Most of the publications reviewed conclude that the recommended dietary reference intake (DRI) should be between 600 and 800 IU/day. Regarding the maximum supplementation dose, the researchers concluded that, for the pediatric population (stated to be 4000 IU/day), there is no evidence to ensure the safety of adolescents.

#### 3.2.4. Dairy

The information on dairy products comes from two systematic reviews [16,18]. In the first review [16], the authors analyzed dairy consumption and its impact on growth, concluding that none of the interventions that included cow’s milk (200 to 500 mL approx.) were proven to have a significant effect on growth. However, relevant differences were observed in the bone mineral content (BMC) and bone mineral density (BMD) of children who consumed cow’s milk (intervention group change (IG%) = 18.46 ± 0.67 vs. control group change (CG%) 16.88 ± 0.6, and IG% = 27 vs. CG% = 24.1).

The second review [18] analyzed dairy consumption and its effects on bone health throughout the life course, drawing on clinical trials and observational studies. This review found that children and adolescents with low or no dairy consumption were at a higher risk of fractures, with lower total BMC and lower z-scores in the femur, hip, and trochanter. Conversely, children who reported higher dairy consumption (500 to 800 mL) had better bone development, as assessed by total BMC (IG = 1490 ± 291 to 1695 ± 317 vs. CG = 1508 ± 167 to 1617 ± 152, *p* = <0.001). Finally, adequate dairy consumption was not associated with total or saturated fat intake nor with excess body weight or fat mass.

Other studies included in this scoping review conclude that dairy consumption is the best option for good health, including bone health, even though they did not directly assess participants’ bone quality. In these studies, the recommended daily intake is 2–3 servings [18,22,23,24,25,27,28].

#### 3.2.5. Beverages

According to the information from six studies included in this scoping review, drinking water should be the primary source of liquid intake among children and adolescents. In addition to water, beverages that are part of a healthy consumption pattern include cow’s milk and, occasionally, 100% fruit or vegetable juices, given their nutritional benefits (up to 120 mL/day) [18,22,23,24,25,27].

##### Sugary Beverages

None of the articles included in this scoping review recommends the consumption of sugary, flavored, carbonated, or caffeinated beverages in children. Additionally, it is suggested that the concept of sugary beverages encompasses not only juices and soft drinks but also smoothies, flavored milk drinks, and sweetened milk with a high content of free sugars, which are reported to be harmful when consumed in excessive amounts [22,27].

##### Vegetable Beverages

As for vegetable beverage consumption, based on the findings of a position paper [24] and a clinical guideline [28], the only vegetable beverage that healthy children and adolescents might consume as a substitute for cow’s milk, under medical indication and nutritional advice, is unsweetened fortified soy milk, because of its nutritional composition, which is mainly due to its content of protein and calcium. Other vegetable drinks are not recommended as substitutes for cow’s milk because they lack certain nutrients.

#### 3.2.6. Sugar

According to a position paper [22] and a clinical guideline [26], a maximum intake of 10% of free sugars in the TEI is recommended for children, although a reduction in free sugar consumption to 5% of the TEI is currently recommended to further minimize sugar intake in children.

According to a position paper [22], total sugars are defined as the sum of naturally occurring sugars in foods, free sugars, and added sugars. The concept of ‘free sugars’ refers to all those foods containing monosaccharides and disaccharides that are added to food or beverages in the manufacturing process and the cooking or preparation of food for consumption, as well as all those sugars that are naturally present in honey, syrups, fruit juices, and fruit juice concentrates. Lastly, the concept of ‘added sugars’ refers to all sugars that are incorporated during the processing or preparation of food (glucose syrups, high-fructose syrups, and isoglucose), except for lactose (which is added to lactose-free milk) and 100% natural fruit or vegetable juices, as well as the fructose naturally present in fruits [22,26].

None of the included studies in our scoping review promoted the consumption of artificial sweeteners in children and adolescents, given the limited information available on potential short- and long-term adverse effects.

#### 3.2.7. Critical Appraisal Within Sources of Evidence

Each article included in this scoping review underwent a critical appraisal of the quality of the evidence. For the systematic reviews, four of the six [16,17,20,21] had critical weaknesses and were therefore classified as critically low confidence; the other two [18,19] were classified as high confidence. According to the AMSTAR tool, the primary issues identified across these studies included the absence of registered protocols for each systematic review. Additionally, one study did not report on the peer-review process for the evaluated articles. Finally, in three systematic reviews, no justification was provided for excluding studies from the review, and no discussion of potential biases in the included studies was provided.

Regarding the position papers, among the four included studies, 75% of the information provided was classified as positive, 14% as neutral or unclear, and 11% as negative [22,23,24,25]. The main methodological issues identified were the absence of clear selection and exclusion criteria for the included studies and the lack of an assessment of their methodological quality. Furthermore, one document did not disclose its funding sources.

Finally, in the four clinical guidelines assessed, the information was unclear, although 60% of the information evaluated with the RIGHT tool was present. The main methodological concerns included insufficient details about the authors who created the guidelines and unclear information about the articles used to formulate the recommendations. Additionally, there was no explicit information on any cost-effectiveness analyses supporting the guidelines’ recommendations. Moreover, none of the guidelines clearly addressed potential conflicts of interest among the authors, nor was it properly reported whether they received any funding [26,27,28,29] (Supplementary Table S4).

## 4. Discussion

This scoping review provides an overview of systematic reviews, position papers, and clinical guidelines published over the past 9 years that address healthy diets for children and adolescents. Six major topics were mapped; the most reported was related to healthy dietary patterns.

Dietary patterns refer to an individual’s eating habits and the range of foods included in their daily diet. There are different types of dietary patterns, which vary by their components (foods) and their effects on metabolic outcomes [30]. A dietary pattern associated with adverse metabolic outcomes is the so-called Western pattern, characterized by high consumption of saturated fats, sugars, and processed foods. This dietary pattern is associated with higher FMI and BMI during childhood [20]. In our scoping review, we found that among the many existing dietary patterns, the MD pattern has been associated with health benefits, including improved body composition (lower FMI and BMI), and may help reduce the risk of childhood obesity [20,31,32,33]. In addition to these, our results show that the MD dietary pattern is considered sustainable [34], as it aligns with the recommendations of the EAT-Lancet Commission for sustainable diets [35].

Despite its many advantages, the MD dietary pattern also has some limitations, as its implementation and evaluation may be subject to methodological biases. There are many instruments to assess this dietary pattern, but they might not be validated for use in children, as reported by Teixeira B. et al. [19]. This variability is an important methodological consideration when extrapolating these findings to clinical practice in children or to health policy.

Another point to highlight is that the evidence pertains only to diets that are not generalizable across different regions of the world. This is due to the dominance of studies conducted in high-income, Western, and Mediterranean countries, which may restrict the applicability of the findings to different sociocultural and economic settings. We identify the lack of evidence from low- and middle-income countries and from diverse cultural contexts as a significant research gap and emphasize it as an important priority for future scoping and systematic reviews.

On the other hand, looking more closely at the components of the diet, specifically micronutrients, we only found information on vitamin D [21]. Based on these results, we found that the current recommendation for vitamin D intake in children and adolescents is 400–600 IU, the same as the recommended DRI for the adult population [21,36]. The recommendation for maximum safety in the adolescent population is 4000 IU; any doses above this recommendation in healthy children and adolescents should be carefully considered, given the lack of reliable and safe information [21].

The Endocrine Society recommends promoting empirical supplementation in children and adolescents by considering consumption of fortified foods or vitamin D supplements; empirical doses are defined as higher than the recommended DRI. This society only issues a recommended dose for the pediatric population, but it is related to the prevention of respiratory tract infections, and this recommendation ranges from 300 to 2000 IU, or an average of 1200 IU per day [37]. Therefore, given the limited and heterogeneous evidence currently available, intake of this vitamin above the recommended DRI should be approached with caution.

It is important to clarify that although other micronutrients were intentionally searched for in this scoping review, only information on vitamin D was found. This is relevant because it may indicate that research on healthy children and adolescents has not been updated in several decades, highlighting a gap in the available information. To consider the complete map, further research on other nutrients, such as iron, zinc, iodine, and B vitamins, is recommended.

Another notable finding from our scoping review is the intake of free sugars in flavored milk beverages. These drinks have become popular and are commonly found in shopping baskets. For this reason, authors such as Fidler Mis et al. [22] suggest modifying the definition of dairy products to differentiate milk from flavored and sweetened dairy drinks. Some authors suggest that those in charge of food shopping for children should reduce the availability of these drinks at home and school lunch to reinforce natural water consumption, which should continue to be the main source of hydration in children and adolescents, followed by cow’s milk [25].

This presents a challenge for parents and families, as children tend to prefer sweet flavors, for example, choosing flavored milk over plain milk. A literature review by Fayet-Moore found that chocolate-flavored milk has the highest palatability rating among children. Some studies indicate that kids drink more flavored milk than plain milk, and when flavored options are not available, they drink less plain milk and, overall, less milk [38]. Conversely, data show that children and teenagers who drink flavored milk are more likely to consume more dairy and follow healthier dietary patterns overall [38,39,40,41,42]. However, flavored milk attracts more criticism than plain milk because of its higher added sugar and calorie content, raising concerns about childhood obesity. Therefore, more studies are needed to assess whether consuming flavored milk poses a long-term health risk or could be a strategy that benefits bone health. This must be carefully evaluated to determine whether the risk of consuming flavored milk outweighs the benefits to children’s and adolescents’ health.

Several studies have addressed the issue of cow’s milk. In children and adolescents, consumption of dairy products derived from cow’s milk is recommended to promote healthy growth and bone development [20,24,26]. The results we found suggest that calcium intake from dairy products benefits total BMC in children and adolescents, regardless of dairy type [16,17]. The Dietary Guidelines for Americans 2020–2025 establish that the recommended dairy products are unsweetened, fat-free, or low-fat (1%) cow’s milk, yogurt, and cheese. Some variants also include lactose-free cow’s milk, unsweetened fortified soy milk, and yogurt [28].

In recent years, the consumption of plant-based beverages by children or adolescents as substitutes for cow’s milk has been increasing. This practice is mainly recommended by parents or caregivers seeking a ‘healthier diet’ or to manage lactose intolerance [43,44]. In any case, and according to our findings, the only vegetable drink that can be recommended because of its similarity to the nutritional content (calcium and protein) of cow’s milk is unsweetened fortified soy milk. According to national health and nutrition organizations, this exchange should take place only under nutritional supervision so that any potential deficiency can be detected in time and appropriate strategies can be developed. Other types of vegetable drinks are not recommended as substitutes for cow’s milk because they are not comparable from a nutritional point of view [24,28].

Other authors, such as Kersting M., have evaluated the replacement of plant-based drinks in the menus of the Optimized Mixed Diet, a balanced eating guide for children in Germany. In their study, they observed that replacing cow’s milk with plant-based beverages reduced overall energy and protein intake and decreased micronutrient intake, including calcium, vitamin B2, vitamin B12, and iodine [45]. On the other hand, a systematic review conducted by Soczynska I. found evidence of lower micronutrient intake and lower height-for-age z-scores in children who consumed plant-based drinks [46]. Based on these findings, it would be advisable that, if choosing to consume plant-based beverages, they be incorporated into a varied and healthy diet, but not as a replacement for cow’s milk, since the evidence suggests potential negative effects on children’s diets and nutrient intake. Additional research is necessary to assess whether there are long-term effects on children’s health and whether consuming higher-quality or more nutritionally valuable plant beverages could make these drinks appropriate substitutes for cow’s milk in children and adolescents.

Based on our literature search, the only study similar to our review was that by Hojsak et al. [47]. This study aimed to provide the most up-to-date information on childhood nutrition and its relationship with certain diseases. Although this study represents a major step forward in updating recommendations on childhood nutrition, it focused on diseases of pediatric interest and not on information on childhood nutrition in a healthy population. The results of this study allowed us to document a large information gap in childhood and adolescent nutrition, underscoring the need to continue updating this important aspect of lifestyle that directly affects health. In our review, we provided an update on childhood nutrition, considering healthy children and adolescents.

The quality of the evidence of the studies included in this scoping review ranged from moderate to low. This is an important factor to bear in mind, since some of the studies assessed used a suitable methodology or execution; however, the information about the protocol registry, description of the financing, or declaration of any conflict of interest by the authors was not clear or sufficient to be able to assign the study a positive rating concerning its adequate control of biases. This information is required under the good methodological practices established by various research groups, such as Cochrane and other health institutions [12,48,49,50]. We recommend treating these results as provisional until confirmed by higher-quality primary studies.

Despite adhering to the relevant methodological considerations for this type of review, our study had some limitations. Owing to the characteristics of a scoping review and the heterogeneity of the information included in this study, it was not possible to carry out a quantitative assessment. Nevertheless, our review sought to address these methodological shortcomings by assessing the methodological quality of the studies and providing more information on the reliability of current dietary recommendations for children and adolescents.

We also noticed that certain information about intuitive eating and body appreciation, parental feeding practices, social media, and body image in adolescents may be relevant to evaluate concerning healthy eating; however, this type of information would require formulating a new research question and conducting an additional literature search, which opens an opportunity gap for new research to enrich the study of nutrition and eating habits in healthy children and adolescents.

One limitation of this study is the inclusion of review articles, which may introduce publication bias due to duplication. This could also result in well-known topics, such as the Mediterranean diet, appearing more frequently as the sole dietary pattern. Additionally, this scoping review considers only articles in Spanish and English, which may limit the scope of available information and underrepresent other regions. As a result, the findings should be viewed with caution.

The information obtained from this scoping review may guide the development of future studies with greater methodological rigor, thereby enabling a more accurate assessment of the current needs of healthy children and adolescents. Additionally, our findings support the application of evidence-based nutritional education strategies and interventions, such as multicomponent and family-centered approaches, with a focus on early childhood. It highlights the significance of culturally tailored approaches and shows how the identified evidence gaps can inform public health policy and future research directions. We also emphasize the need to incorporate behavioral science perspectives, considering motivational and environmental factors.

## 5. Conclusions

This scoping review provides updated information on dietary recommendations for healthy children and adolescents. We found that the current information is largely focused on dietary patterns. However, these patterns need to be adapted to different populations, socio-cultural contexts, and sustainability requirements. Additionally, directions for future interdisciplinary research suggest that a comprehensive understanding of pediatric nutrition ultimately depends on integrating behavioral and psychological sciences with evidence regarding dietary patterns.

In addition, it is necessary to consider the quality of the evidence, which ranged from moderate to low. Overall, the quality of the evidence is limited, so this scoping review’s findings should be interpreted cautiously. This also highlights the knowledge gap that remains and warrants further investigation to better understand the diet landscape in healthy children and adolescents.

This scoping review provides updated evidence on dietary recommendations for healthy children and adolescents. Despite significant progress in pediatric nutrition, important research gaps remain. Ensuring that healthy children and adolescents receive evidence-based nutritional guidance must remain a priority in both research and public health policy.

## Figures and Tables

**Table 1:** Description of the studies on child nutrition of healthy children and adolescents published between 2016 and 2024.

Study Information	Nutrient/Component	*n*	Age	Country or Region	Related Objective to Child Nutrition	Conclusions Reported by the Authors
**Systematic reviews**						
Te Morenga and Montez (2017) [17]	Fatty acids (SFA and TFA)	2.430	2–19 years	Global	Effect of SFA and TFA intake in children	Better cholesterol outcomes with SFA < 10% of TEI
de Lamas et al. (2019) [16]	Dairy	3.895	Mean age: 9.95 years; Age range: 3–18 years.	Global	Dairy products, linear growth, and the BMC	BMC rises with the addition of dairy products to the regular diet.
Patseadou and Haller (2020) [21]	Vitamin D	Not specified	10–19 years	Global	Recommendations on vitamin D in adolescents	Education about risk factors associated with low VD levels is essential. Regular outdoor physical activity and careful sun exposure are of utmost importance.
Boushey et al. (2020) [20]	Dietary patterns	Not specified	All ages	Australia, Portugal, Netherlands, United Kingdom, United States	Dietary patterns, growth, body composition, and risk of being overweight or obese	Unhealthy eating habits are linked to higher FMI and BMI during adolescence.
Wallace et al. (2021) [18]	Dairy	7685 *	All ages (children and adolescents from 3 to 19 years of age)	Global	Role of dairy products and bone health in a lifetime	Daily intake of low-fat or fat-free dairy is associated with a better total BMD.
Teixeira et al. (2022) [19]	Sustainable dietary patterns.	329,898	2–17 years	Global	Identify, characterize, and assess adherence to healthy and sustainable dietary patterns	The MD pattern is sustainable and the most studied in pediatrics. Associated with better metabolic outcomes. Results were evaluated in a heterogeneous manner.
**Position papers**						
Fiddle Mis et al. (2017) [22]	Sugars, sweeteners, and flavored drinks	Not specified	Pediatric age	Global	Recommendations on the consumption of sugars and sugar-sweetened beverages in children and adolescents	Smoothies and flavored or sweetened milks contain free sugars, although they are not considered sugary drinks.
Dereń et al. (2019) [25]	Flavored or sugary drinks	Not specified	Pediatric age	Global	Risks associated with the consumption of flavored beverages in infants, children, and adolescents	Sugary drinks should not be available in schools. Avoid using flavored drinks to please children.
Lott et al. (2021) [24]	Drinks	Not specified	0–5 years	USA	Oral health and beverage consumption	Consumption of plain water during the first five years of age reduces excessive intake of sugar and saturated fat.
Hoelscher et al. (2022) [23]	Prevention of overweight or obesity	Not specified	Pediatric age	Global	Interventions aimed at preventing childhood overweight or obesity	The variability in dietary patterns is influenced by multiple factors, such as NSS, seasonality, culture, religion, and others.
**Clinical guidelines**						
Mexican Social Security Institute (IMSS) 2016 [27]	Healthy eating patterns	Not specified	All ages, including pediatric age	Mexico	Dietary patterns and prevention of chronic degenerative diseases	No reported findings
World Health Organization (WHO) 2017 [26]	Child’s Health	Not specified	Pediatric age	Global	WHO’s recommendations on child health	No reported findings
United States Department of Agriculture (2020) [28]	Healthy diet	Not specified	All ages, including pediatric age	USA	Promoting health to reduce the risk of diet-related chronic diseases	No reported findings
World Health Organization (WHO) 2022 [29]	Healthy diet and sustainability	Not specified	All ages, including pediatric age	Global	WHO’s recommendations to address different health problems	Consuming locally grown food.

* Estimated by the reviewers of this study, the total number of children and adolescents in the study was not reported. Abbreviations: SFAs = saturated fatty acids, TFAs = trans fatty acids, TEI = total energy intake, BMC = bone mineral content, VD = Vitamin D, FMI = fat mass index, BMI = body mass index, BMD = bone mineral density, MD = Mediterranean diet, NSS = socioeconomic level, USA = United States of America.

## Data Availability

The datasets generated and analyzed during this study are not publicly available due to legal and ethical reasons but can be accessed from the corresponding author upon reasonable request.

## Supplementary Materials

The following supporting information can be downloaded at: https://www.mdpi.com/article/10.3390/nu18101578/s1, Table S1: PEOS. Table S2: Search terms. Table S3: Excluded articles and reasons. Table S4: Quality appraisal of included studies.

## Abbreviations

The following abbreviations are used in this manuscript:

MD	Mediterranean diet
BMI	Body mass index
WHO	World Health Organization
SFAs	Saturated fatty acids
TFAs	Trans fatty acids
TEI	Total energy intake
DRI	Dietary reference intake
VD	Vitamin D
IU	International units
BMC	Bone mineral content
BMD	Bone mineral density
SES	Socioeconomic status
