# Supplementary material for: Mapping Current Evidence and Research Gaps in Nutrition for Healthy Children and Adolescents: A Scoping Review
Source: Nutrients. 2026 May 15;18(10):1578. doi: 10.3390/nu18101578 (PMC13210225; doi:10.3390/nu18101578)
Supplement: Supplementary file 1 [file nutrients-18-01578-s001.zip › nutrients-4294432-supplementary.pdf]

Article title: Mapping Current Evidence and Research Gaps in Nutrition for Healthy Children and Adolescents: A Scoping Review

Diana Montiel-Ojeda [1], Lucía Méndez-Sánchez [2], Desiree Lopez-Gonzalez [1,*] and Patricia Clark [1,*]

[1] Clinical Epidemiology Research Unit, Children's Hospital of Mexico Federico Gomez, Faculty of Medicine, National Autonomous University of Mexico UNAM, Mexico City 06720, Mexico; diana-montiel@facmed.unam.mx

[2] Cochrane Mexico UNAM Associated Group, Library and Information System, Faculty of Medicine, National Autonomous University of Mexico UNAM, Mexico City 04510, Mexico; lmendez@facmed.unam.mx

* Correspondence: dradesireelopez@gmail.com (D.L.-G.); clark@unam.mx (P.C.); Tel: +52-55-5228-9917 (ext. 2371) (D.L.-G. & P.C.)

Supplementary Table S1. PEOS

Population	Children and adolescents between 2 and 19 years old
Exposition	Dietary guidelines, food-based dietary guidelines, diet
Outcome	Healthy nutrition in children with no diagnosed chronic disease, acute illness, or nutritional disorder at the time of assessment.
Studies	Systematic reviews, position papers, and clinical practice guidelines

Supplementary Table S2. Search terms.

Search: **child adolescent dietary guidelines** Filters: **Systematic Review, from 2016 - 2024**
(("child"[MeSH Terms] OR "child"[All Fields] OR "children"[All Fields] OR "child s"[All Fields] OR "children s"[All Fields] OR "childrens"[All Fields] OR "childs"[All Fields]) AND ("adolescences"[All Fields] OR "adolescency"[All Fields] OR "adolescent"[MeSH Terms] OR "adolescent"[All Fields] OR "adolescence"[All Fields] OR "adolescents"[All Fields] OR "adolescent s"[All Fields]) AND ("nutrition policy"[MeSH Terms] OR ("nutrition"[All Fields] AND "policy"[All Fields]) OR "nutrition policy"[All Fields] OR ("dietary"[All Fields] AND "guidelines"[All Fields]) OR "dietary guidelines"[All Fields])) AND ((systematicreview[Filter]) AND (2016:2022[pdat]))
Search: **child adolescent dietary guidelines vitamins** Filters: **Systematic Review, from 2016 - 2024**
(("child"[MeSH Terms] OR "child"[All Fields] OR "children"[All Fields] OR "child s"[All Fields] OR "children s"[All Fields] OR "childrens"[All Fields] OR "childs"[All Fields]) AND ("adolescences"[All Fields] OR "adolescency"[All Fields] OR "adolescent"[MeSH Terms] OR "adolescent"[All Fields] OR "adolescence"[All Fields] OR "adolescents"[All Fields] OR "adolescent s"[All Fields]) AND ("nutrition policy"[MeSH Terms] OR ("nutrition"[All Fields] AND "policy"[All Fields]) OR "nutrition policy"[All Fields] OR ("dietary"[All Fields] AND "guidelines"[All Fields]) OR "dietary guidelines"[All Fields]) AND ("vitamin s"[All Fields] OR "vitamine"[All Fields] OR "vitamines"[All Fields] OR "vitamins"[Pharmacological Action] OR "vitamins"[MeSH Terms] OR "vitamins"[All Fields] OR "vitamin"[All Fields])) AND ((systematicreview[Filter]) AND (2016:2022[pdat]))
Search: **child adolescent dietary guidelines minerals** Filters: **Systematic Review, from 2016 - 2024**
(("child"[MeSH Terms] OR "child"[All Fields] OR "children"[All Fields] OR "child s"[All Fields] OR "children s"[All Fields] OR "childrens"[All Fields] OR "childs"[All Fields]) AND ("adolescences"[All Fields] OR "adolescency"[All Fields] OR "adolescent"[MeSH Terms] OR "adolescent"[All Fields] OR "adolescence"[All Fields] OR "adolescents"[All Fields] OR "adolescent s"[All Fields]) AND ("nutrition policy"[MeSH Terms] OR ("nutrition"[All Fields] AND "policy"[All Fields]) OR "nutrition policy"[All Fields] OR ("dietary"[All Fields] AND "guidelines"[All Fields]) OR "dietary guidelines"[All Fields]) AND ("calcification, physiologic"[MeSH Terms] OR ("calcification"[All Fields] AND "physiologic"[All Fields]) OR "physiologic calcification"[All Fields] OR "mineralization"[All Fields] OR "mineral s"[All Fields] OR "mineralisable"[All Fields] OR "mineralisation"[All Fields] OR "mineralisations"[All Fields] OR "mineralise"[All Fields] OR "mineralised"[All Fields] OR "mineralising"[All Fields] OR "mineralizations"[All Fields] OR "mineralize"[All Fields] OR "mineralized"[All Fields] OR "mineralizer"[All Fields] OR "mineralizers"[All Fields] OR "mineralizes"[All Fields] OR "mineralizing"[All Fields] OR "minerals"[MeSH Terms] OR "minerals"[All Fields] OR "mineral"[All Fields])) AND ((systematicreview[Filter]) AND (2016:2022[pdat]))
Search: **child adolescent dietary guidelines sugar** Filters: **Systematic Review, from 2016 - 2024**

(("child"[MeSH Terms] OR "child"[All Fields] OR "children"[All Fields] OR "child s"[All Fields] OR "children s"[All Fields] OR "childrens"[All Fields] OR "childs"[All Fields]) AND ("adolescences"[All Fields] OR "adolescency"[All Fields] OR "adolescent"[MeSH Terms] OR "adolescent"[All Fields] OR "adolescence"[All Fields] OR "adolescents"[All Fields] OR "adolescent s"[All Fields]) AND ("nutrition policy"[MeSH Terms] OR ("nutrition"[All Fields] AND "policy"[All Fields]) OR "nutrition policy"[All Fields] OR ("dietary"[All Fields] AND "guidelines"[All Fields]) OR "dietary guidelines"[All Fields]) AND ("sugar s"[All Fields] OR "sugared"[All Fields] OR "sugars"[MeSH Terms] OR "sugars"[All Fields] OR "sugar"[All Fields])) AND ((systematicreview[Filter]) AND (2016:2022[pdat]))
Search: **child adolescent dietary guidelines sweeteners** Filters: **Systematic Review, from 2016 - 2024**
(("child"[MeSH Terms] OR "child"[All Fields] OR "children"[All Fields] OR "child s"[All Fields] OR "children s"[All Fields] OR "childrens"[All Fields] OR "childs"[All Fields]) AND ("adolescences"[All Fields] OR "adolescency"[All Fields] OR "adolescent"[MeSH Terms] OR "adolescent"[All Fields] OR "adolescence"[All Fields] OR "adolescents"[All Fields] OR "adolescent s"[All Fields]) AND ("nutrition policy"[MeSH Terms] OR ("nutrition"[All Fields] AND "policy"[All Fields]) OR "nutrition policy"[All Fields] OR ("dietary"[All Fields] AND "guidelines"[All Fields]) OR "dietary guidelines"[All Fields]) AND ("sweeten"[All Fields] OR "sweetened"[All Fields] OR "sweetening agents"[Pharmacological Action] OR "sweetening agents"[MeSH Terms] OR ("sweetening"[All Fields] AND "agents"[All Fields]) OR "sweetening agents"[All Fields] OR "sweetener"[All Fields] OR "sweeteners"[All Fields] OR "sweetening"[All Fields])) AND ((systematicreview[Filter]) AND (2016:2022[pdat]))
Search: **child adolescent dietary guidelines vegetarian** Filters: **Systematic Review, from 2016 - 2024**
(("child"[MeSH Terms] OR "child"[All Fields] OR "children"[All Fields] OR "child s"[All Fields] OR "children s"[All Fields] OR "childrens"[All Fields] OR "childs"[All Fields]) AND ("adolescences"[All Fields] OR "adolescency"[All Fields] OR "adolescent"[MeSH Terms] OR "adolescent"[All Fields] OR "adolescence"[All Fields] OR "adolescents"[All Fields] OR "adolescent s"[All Fields]) AND ("nutrition policy"[MeSH Terms] OR ("nutrition"[All Fields] AND "policy"[All Fields]) OR "nutrition policy"[All Fields] OR ("dietary"[All Fields] AND "guidelines"[All Fields]) OR "dietary guidelines"[All Fields]) AND ("diet, vegetarian"[MeSH Terms] OR ("diet"[All Fields] AND "vegetarian"[All Fields]) OR "vegetarian diet"[All Fields] OR "vegetarianism"[All Fields] OR "vegetarians"[MeSH Terms] OR "vegetarians"[All Fields] OR "vegetarian"[All Fields])) AND ((systematicreview[Filter]) AND (2016:2022[pdat]))
Search: **child adolescent dietary guidelines dairy** Filters: **Systematic Review, from 2016 - 2024**

(("child"[MeSH Terms] OR "child"[All Fields] OR "children"[All Fields] OR "child s"[All Fields] OR "children s"[All Fields] OR "childrens"[All Fields] OR "childs"[All Fields]) AND ("adolescences"[All Fields] OR "adolescency"[All Fields] OR "adolescent"[MeSH Terms] OR "adolescent"[All Fields] OR "adolescence"[All Fields] OR "adolescents"[All Fields] OR "adolescent s"[All Fields]) AND ("nutrition policy"[MeSH Terms] OR ("nutrition"[All Fields] AND "policy"[All Fields]) OR "nutrition policy"[All Fields] OR ("dietary"[All Fields] AND "guidelines"[All Fields]) OR "dietary guidelines"[All Fields]) AND ("dairies"[All Fields] OR "dairy"[All Fields] OR "dairy s"[All Fields] OR "dairying"[MeSH Terms] OR "dairying"[All Fields])) AND ((systematicreview[Filter]) AND (2016:2022[pdat]))
Search: **child adolescent food-based dietary guidelines** Filters: **Systematic Review, from 2016 - 2024**
(("child"[MeSH Terms] OR "child"[All Fields] OR "children"[All Fields] OR "child s"[All Fields] OR "children s"[All Fields] OR "childrens"[All Fields] OR "childs"[All Fields]) AND ("adolescences"[All Fields] OR "adolescency"[All Fields] OR "adolescent"[MeSH Terms] OR "adolescent"[All Fields] OR "adolescence"[All Fields] OR "adolescents"[All Fields] OR "adolescent s"[All Fields]) AND "food-based"[All Fields] AND ("nutrition policy"[MeSH Terms] OR ("nutrition"[All Fields] AND "policy"[All Fields]) OR "nutrition policy"[All Fields] OR ("dietary"[All Fields] AND "guidelines"[All Fields]) OR "dietary guidelines"[All Fields])) AND ((systematicreview[Filter]) AND (2016:2022[pdat]))
Search: **(child adolescent dietary guidelines) AND (position paper)** Filters: **from 2016 - 2024**
(("child"[MeSH Terms] OR "child"[All Fields] OR "children"[All Fields] OR "child s"[All Fields] OR "children s"[All Fields] OR "childrens"[All Fields] OR "childs"[All Fields]) AND ("adolescences"[All Fields] OR "adolescency"[All Fields] OR "adolescent"[MeSH Terms] OR "adolescent"[All Fields] OR "adolescence"[All Fields] OR "adolescents"[All Fields] OR "adolescent s"[All Fields]) AND ("nutrition policy"[MeSH Terms] OR ("nutrition"[All Fields] AND "policy"[All Fields]) OR "nutrition policy"[All Fields] OR ("dietary"[All Fields] AND "guidelines"[All Fields]) OR "dietary guidelines"[All Fields]) AND (("patient positioning"[MeSH Terms] OR ("patient"[All Fields] AND "positioning"[All Fields]) OR "patient positioning"[All Fields] OR "positioning"[All Fields] OR "position"[All Fields] OR "position s"[All Fields] OR "positional"[All Fields] OR "positioned"[All Fields] OR "positionings"[All Fields] OR "positions"[All Fields]) AND ("paper"[MeSH Terms] OR "paper"[All Fields] OR "papers"[All Fields] OR "paper s"[All Fields]))) AND (2016:2022[pdat])
Search: **(child adolescent dietary guidelines vitamins) AND (position paper)** Filters: **from 2016 - 2024**

(("child"[MeSH Terms] OR "child"[All Fields] OR "children"[All Fields] OR "child s"[All Fields] OR "children s"[All Fields] OR "childrens"[All Fields] OR "childs"[All Fields]) AND ("adolescences"[All Fields] OR "adolescency"[All Fields] OR "adolescent"[MeSH Terms] OR "adolescent"[All Fields] OR "adolescence"[All Fields] OR "adolescents"[All Fields] OR "adolescent s"[All Fields]) AND ("nutrition policy"[MeSH Terms] OR ("nutrition"[All Fields] AND "policy"[All Fields]) OR "nutrition policy"[All Fields] OR ("dietary"[All Fields] AND "guidelines"[All Fields]) OR "dietary guidelines"[All Fields]) AND ("vitamin s"[All Fields] OR "vitamine"[All Fields] OR "vitamines"[All Fields] OR "vitamins"[Pharmacological Action] OR "vitamins"[MeSH Terms] OR "vitamins"[All Fields] OR "vitamin"[All Fields]) AND (("patient positioning"[MeSH Terms] OR ("patient"[All Fields] AND "positioning"[All Fields]) OR "patient positioning"[All Fields] OR "positioning"[All Fields] OR "position"[All Fields] OR "position s"[All Fields] OR "positional"[All Fields] OR "positioned"[All Fields] OR "positionings"[All Fields] OR "positions"[All Fields]) AND ("paper"[MeSH Terms] OR "paper"[All Fields] OR "papers"[All Fields] OR "paper s"[All Fields]))) AND (2016:2022[pdat])
Search: **(child adolescent dietary guidelines minerals) AND (position paper)** Filters: **from 2016 - 2024**
(("child"[MeSH Terms] OR "child"[All Fields] OR "children"[All Fields] OR "child s"[All Fields] OR "children s"[All Fields] OR "childrens"[All Fields] OR "childs"[All Fields]) AND ("adolescences"[All Fields] OR "adolescency"[All Fields] OR "adolescent"[MeSH Terms] OR "adolescent"[All Fields] OR "adolescence"[All Fields] OR "adolescents"[All Fields] OR "adolescent s"[All Fields]) AND ("nutrition policy"[MeSH Terms] OR ("nutrition"[All Fields] AND "policy"[All Fields]) OR "nutrition policy"[All Fields] OR ("dietary"[All Fields] AND "guidelines"[All Fields]) OR "dietary guidelines"[All Fields]) AND ("calcification, physiologic"[MeSH Terms] OR ("calcification"[All Fields] AND "physiologic"[All Fields]) OR "physiologic calcification"[All Fields] OR "mineralization"[All Fields] OR "mineral s"[All Fields] OR "mineralisable"[All Fields] OR "mineralisation"[All Fields] OR "mineralisations"[All Fields] OR "mineralise"[All Fields] OR "mineralised"[All Fields] OR "mineralising"[All Fields] OR "mineralizations"[All Fields] OR "mineralize"[All Fields] OR "mineralized"[All Fields] OR "mineralizer"[All Fields] OR "mineralizers"[All Fields] OR "mineralizes"[All Fields] OR "mineralizing"[All Fields] OR "minerals"[MeSH Terms] OR "minerals"[All Fields] OR "mineral"[All Fields]) AND (("patient positioning"[MeSH Terms] OR ("patient"[All Fields] AND "positioning"[All Fields]) OR "patient positioning"[All Fields] OR "positioning"[All Fields] OR "position"[All Fields] OR "position s"[All Fields] OR "positional"[All Fields] OR "positioned"[All Fields] OR "positionings"[All Fields] OR "positions"[All Fields]) AND ("paper"[MeSH Terms] OR "paper"[All Fields] OR "papers"[All Fields] OR "paper s"[All Fields]))) AND (2016:2022[pdat])
Search: **(child adolescent dietary guidelines sugar) AND (position paper)** Filters: **from 2016 - 2024**

(("child"[MeSH Terms] OR "child"[All Fields] OR "children"[All Fields] OR "child s"[All Fields] OR "children s"[All Fields] OR "childrens"[All Fields] OR "childs"[All Fields]) AND ("adolescences"[All Fields] OR "adolescency"[All Fields] OR "adolescent"[MeSH Terms] OR "adolescent"[All Fields] OR "adolescence"[All Fields] OR "adolescents"[All Fields] OR "adolescent s"[All Fields]) AND ("nutrition policy"[MeSH Terms] OR ("nutrition"[All Fields] AND "policy"[All Fields]) OR "nutrition policy"[All Fields] OR ("dietary"[All Fields] AND "guidelines"[All Fields]) OR "dietary guidelines"[All Fields]) AND ("sugar s"[All Fields] OR "sugared"[All Fields] OR "sugars"[MeSH Terms] OR "sugars"[All Fields] OR "sugar"[All Fields]) AND (("patient positioning"[MeSH Terms] OR ("patient"[All Fields] AND "positioning"[All Fields]) OR "patient positioning"[All Fields] OR "positioning"[All Fields] OR "position"[All Fields] OR "position s"[All Fields] OR "positional"[All Fields] OR "positioned"[All Fields] OR "positionings"[All Fields] OR "positions"[All Fields]) AND ("paper"[MeSH Terms] OR "paper"[All Fields] OR "papers"[All Fields] OR "paper s"[All Fields]))) AND (2016:2022[pdat])
Search: **(child adolescent dietary guidelines sweeteners) AND (position paper)** Filters: **from 2016 - 2024**
(("child"[MeSH Terms] OR "child"[All Fields] OR "children"[All Fields] OR "child s"[All Fields] OR "children s"[All Fields] OR "childrens"[All Fields] OR "childs"[All Fields]) AND ("adolescences"[All Fields] OR "adolescency"[All Fields] OR "adolescent"[MeSH Terms] OR "adolescent"[All Fields] OR "adolescence"[All Fields] OR "adolescents"[All Fields] OR "adolescent s"[All Fields]) AND ("nutrition policy"[MeSH Terms] OR ("nutrition"[All Fields] AND "policy"[All Fields]) OR "nutrition policy"[All Fields] OR ("dietary"[All Fields] AND "guidelines"[All Fields]) OR "dietary guidelines"[All Fields]) AND ("sweeten"[All Fields] OR "sweetened"[All Fields] OR "sweetening agents"[Pharmacological Action] OR "sweetening agents"[MeSH Terms] OR ("sweetening"[All Fields] AND "agents"[All Fields]) OR "sweetening agents"[All Fields] OR "sweetener"[All Fields] OR "sweeteners"[All Fields] OR "sweetening"[All Fields]) AND (("patient positioning"[MeSH Terms] OR ("patient"[All Fields] AND "positioning"[All Fields]) OR "patient positioning"[All Fields] OR "positioning"[All Fields] OR "position"[All Fields] OR "position s"[All Fields] OR "positional"[All Fields] OR "positioned"[All Fields] OR "positionings"[All Fields] OR "positions"[All Fields]) AND ("paper"[MeSH Terms] OR "paper"[All Fields] OR "papers"[All Fields] OR "paper s"[All Fields]))) AND (2016:2022[pdat])
Search: **(child adolescent dietary guidelines vegetarian) AND (position paper)** Filters: **from 2016 - 2024**

(("child"[MeSH Terms] OR "child"[All Fields] OR "children"[All Fields] OR "child s"[All Fields] OR "children s"[All Fields] OR "childrens"[All Fields] OR "childs"[All Fields]) AND ("adolescences"[All Fields] OR "adolescency"[All Fields] OR "adolescent"[MeSH Terms] OR "adolescent"[All Fields] OR "adolescence"[All Fields] OR "adolescents"[All Fields] OR "adolescent s"[All Fields]) AND ("nutrition policy"[MeSH Terms] OR ("nutrition"[All Fields] AND "policy"[All Fields]) OR "nutrition policy"[All Fields] OR ("dietary"[All Fields] AND "guidelines"[All Fields]) OR "dietary guidelines"[All Fields]) AND ("diet, vegetarian"[MeSH Terms] OR ("diet"[All Fields] AND "vegetarian"[All Fields]) OR "vegetarian diet"[All Fields] OR "vegetarianism"[All Fields] OR "vegetarians"[MeSH Terms] OR "vegetarians"[All Fields] OR "vegetarian"[All Fields]) AND (("patient positioning"[MeSH Terms] OR ("patient"[All Fields] AND "positioning"[All Fields]) OR "patient positioning"[All Fields] OR "positioning"[All Fields] OR "position"[All Fields] OR "position s"[All Fields] OR "positional"[All Fields] OR "positioned"[All Fields] OR "positionings"[All Fields] OR "positions"[All Fields]) AND ("paper"[MeSH Terms] OR "paper"[All Fields] OR "papers"[All Fields] OR "paper s"[All Fields]))) AND (2016:2022[pdat])
Search: **(child adolescent dietary guidelines dairy) AND (position paper)** Filters: **from 2016 - 2024**
(("child"[MeSH Terms] OR "child"[All Fields] OR "children"[All Fields] OR "child s"[All Fields] OR "children s"[All Fields] OR "childrens"[All Fields] OR "childs"[All Fields]) AND ("adolescences"[All Fields] OR "adolescency"[All Fields] OR "adolescent"[MeSH Terms] OR "adolescent"[All Fields] OR "adolescence"[All Fields] OR "adolescents"[All Fields] OR "adolescent s"[All Fields]) AND ("nutrition policy"[MeSH Terms] OR ("nutrition"[All Fields] AND "policy"[All Fields]) OR "nutrition policy"[All Fields] OR ("dietary"[All Fields] AND "guidelines"[All Fields]) OR "dietary guidelines"[All Fields]) AND ("dairies"[All Fields] OR "dairy"[All Fields] OR "dairy s"[All Fields] OR "dairying"[MeSH Terms] OR "dairying"[All Fields]) AND (("patient positioning"[MeSH Terms] OR ("patient"[All Fields] AND "positioning"[All Fields]) OR "patient positioning"[All Fields] OR "positioning"[All Fields] OR "position"[All Fields] OR "position s"[All Fields] OR "positional"[All Fields] OR "positioned"[All Fields] OR "positionings"[All Fields] OR "positions"[All Fields]) AND ("paper"[MeSH Terms] OR "paper"[All Fields] OR "papers"[All Fields] OR "paper s"[All Fields]))) AND (2016:2022[pdat])
Search: **(child adolescent food-based dietary guidelines) AND (position paper)** Filters: **from 2016 - 2024**
(("child"[MeSH Terms] OR "child"[All Fields] OR "children"[All Fields] OR "child s"[All Fields] OR "children s"[All Fields] OR "childrens"[All Fields] OR "childs"[All Fields]) AND ("adolescences"[All Fields] OR "adolescency"[All Fields] OR "adolescent"[MeSH Terms] OR "adolescent"[All Fields] OR "adolescence"[All Fields] OR "adolescents"[All Fields] OR "adolescent s"[All Fields]) AND "food-based"[All Fields] AND ("nutrition policy"[MeSH Terms] OR ("nutrition"[All Fields] AND "policy"[All Fields]) OR "nutrition policy"[All Fields] OR ("dietary"[All Fields] AND "guidelines"[All Fields]) OR "dietary guidelines"[All Fields]) AND (("patient positioning"[MeSH Terms] OR ("patient"[All Fields] AND "positioning"[All Fields]) OR "patient positioning"[All Fields] OR "positioning"[All Fields] OR "position"[All Fields] OR "position s"[All Fields] OR "positional"[All Fields] OR "positioned"[All Fields] OR "positionings"[All Fields] OR "positions"[All Fields]) AND ("paper"[MeSH Terms] OR "paper"[All Fields] OR "papers"[All Fields] OR "paper s"[All Fields]))) AND (2016:2022[pdat])

Search: **child adolescent dietary guidelines** Filters: **Guideline, Practice Guideline, from 2016 - 2024**
(("child"[MeSH Terms] OR "child"[All Fields] OR "children"[All Fields] OR "child s"[All Fields] OR "children s"[All Fields] OR "childrens"[All Fields] OR "childs"[All Fields]) AND ("adolescences"[All Fields] OR "adolescency"[All Fields] OR "adolescent"[MeSH Terms] OR "adolescent"[All Fields] OR "adolescence"[All Fields] OR "adolescents"[All Fields] OR "adolescent s"[All Fields]) AND ("nutrition policy"[MeSH Terms] OR ("nutrition"[All Fields] AND "policy"[All Fields]) OR "nutrition policy"[All Fields] OR ("dietary"[All Fields] AND "guidelines"[All Fields]) OR "dietary guidelines"[All Fields])) AND ((guideline[Filter] OR practiceguideline[Filter]) AND (2016:2022[pdat]))
Search: **child adolescent dietary guidelines vitamins** Filters: **Guideline, Practice Guideline, from 2016 - 2024**
(("child"[MeSH Terms] OR "child"[All Fields] OR "children"[All Fields] OR "child s"[All Fields] OR "children s"[All Fields] OR "childrens"[All Fields] OR "childs"[All Fields]) AND ("adolescences"[All Fields] OR "adolescency"[All Fields] OR "adolescent"[MeSH Terms] OR "adolescent"[All Fields] OR "adolescence"[All Fields] OR "adolescents"[All Fields] OR "adolescent s"[All Fields]) AND ("nutrition policy"[MeSH Terms] OR ("nutrition"[All Fields] AND "policy"[All Fields]) OR "nutrition policy"[All Fields] OR ("dietary"[All Fields] AND "guidelines"[All Fields]) OR "dietary guidelines"[All Fields]) AND ("vitamin s"[All Fields] OR "vitamine"[All Fields] OR "vitamines"[All Fields] OR "vitamins"[Pharmacological Action] OR "vitamins"[MeSH Terms] OR "vitamins"[All Fields] OR "vitamin"[All Fields])) AND ((guideline[Filter] OR practiceguideline[Filter]) AND (2016:2022[pdat]))
Search: **child adolescent dietary guidelines minerals** Filters: **Guideline, Practice Guideline, from 2016 - 2024**
(("child"[MeSH Terms] OR "child"[All Fields] OR "children"[All Fields] OR "child s"[All Fields] OR "children s"[All Fields] OR "childrens"[All Fields] OR "childs"[All Fields]) AND ("adolescences"[All Fields] OR "adolescency"[All Fields] OR "adolescent"[MeSH Terms] OR "adolescent"[All Fields] OR "adolescence"[All Fields] OR "adolescents"[All Fields] OR "adolescent s"[All Fields]) AND ("nutrition policy"[MeSH Terms] OR ("nutrition"[All Fields] AND "policy"[All Fields]) OR "nutrition policy"[All Fields] OR ("dietary"[All Fields] AND "guidelines"[All Fields]) OR "dietary guidelines"[All Fields]) AND ("calcification, physiologic"[MeSH Terms] OR ("calcification"[All Fields] AND "physiologic"[All Fields]) OR "physiologic calcification"[All Fields] OR "mineralization"[All Fields] OR "mineral s"[All Fields] OR "mineralisable"[All Fields] OR "mineralisation"[All Fields] OR "mineralisations"[All Fields] OR "mineralise"[All Fields] OR "mineralised"[All Fields] OR "mineralising"[All Fields] OR "mineralizations"[All Fields] OR "mineralize"[All Fields] OR "mineralized"[All Fields] OR "mineralizer"[All Fields] OR "mineralizers"[All Fields] OR "mineralizes"[All Fields] OR "mineralizing"[All Fields] OR "minerals"[MeSH Terms] OR "minerals"[All Fields] OR "mineral"[All Fields])) AND ((guideline[Filter] OR practiceguideline[Filter]) AND (2016:2022[pdat]))
Search: **child adolescent dietary guidelines sugar** Filters: **Guideline, Practice Guideline, from 2016 - 2024**

(("child"[MeSH Terms] OR "child"[All Fields] OR "children"[All Fields] OR "child s"[All Fields] OR "children s"[All Fields] OR "childrens"[All Fields] OR "childs"[All Fields]) AND ("adolescences"[All Fields] OR "adolescency"[All Fields] OR "adolescent"[MeSH Terms] OR "adolescent"[All Fields] OR "adolescence"[All Fields] OR "adolescents"[All Fields] OR "adolescent s"[All Fields]) AND ("nutrition policy"[MeSH Terms] OR ("nutrition"[All Fields] AND "policy"[All Fields]) OR "nutrition policy"[All Fields] OR ("dietary"[All Fields] AND "guidelines"[All Fields]) OR "dietary guidelines"[All Fields]) AND ("sugar s"[All Fields] OR "sugared"[All Fields] OR "sugars"[MeSH Terms] OR "sugars"[All Fields] OR "sugar"[All Fields])) AND ((guideline[Filter] OR practiceguideline[Filter]) AND (2016:2022[pdat]))
Search: **child adolescent dietary guidelines sweeteners** Filters: **Guideline, Practice Guideline, from 2016 - 2024**
(("child"[MeSH Terms] OR "child"[All Fields] OR "children"[All Fields] OR "child s"[All Fields] OR "children s"[All Fields] OR "childrens"[All Fields] OR "childs"[All Fields]) AND ("adolescences"[All Fields] OR "adolescency"[All Fields] OR "adolescent"[MeSH Terms] OR "adolescent"[All Fields] OR "adolescence"[All Fields] OR "adolescents"[All Fields] OR "adolescent s"[All Fields]) AND ("nutrition policy"[MeSH Terms] OR ("nutrition"[All Fields] AND "policy"[All Fields]) OR "nutrition policy"[All Fields] OR ("dietary"[All Fields] AND "guidelines"[All Fields]) OR "dietary guidelines"[All Fields]) AND ("sweeten"[All Fields] OR "sweetened"[All Fields] OR "sweetening agents"[Pharmacological Action] OR "sweetening agents"[MeSH Terms] OR ("sweetening"[All Fields] AND "agents"[All Fields]) OR "sweetening agents"[All Fields] OR "sweetener"[All Fields] OR "sweeteners"[All Fields] OR "sweetening"[All Fields])) AND ((guideline[Filter] OR practiceguideline[Filter]) AND (2016:2022[pdat]))
Search: **child adolescent dietary guidelines vegetarian** Filters: **Guideline, Practice Guideline, from 2016 - 2024**
(("child"[MeSH Terms] OR "child"[All Fields] OR "children"[All Fields] OR "child s"[All Fields] OR "children s"[All Fields] OR "childrens"[All Fields] OR "childs"[All Fields]) AND ("adolescences"[All Fields] OR "adolescency"[All Fields] OR "adolescent"[MeSH Terms] OR "adolescent"[All Fields] OR "adolescence"[All Fields] OR "adolescents"[All Fields] OR "adolescent s"[All Fields]) AND ("nutrition policy"[MeSH Terms] OR ("nutrition"[All Fields] AND "policy"[All Fields]) OR "nutrition policy"[All Fields] OR ("dietary"[All Fields] AND "guidelines"[All Fields]) OR "dietary guidelines"[All Fields]) AND ("diet, vegetarian"[MeSH Terms] OR ("diet"[All Fields] AND "vegetarian"[All Fields]) OR "vegetarian diet"[All Fields] OR "vegetarianism"[All Fields] OR "vegetarians"[MeSH Terms] OR "vegetarians"[All Fields] OR "vegetarian"[All Fields])) AND ((guideline[Filter] OR practiceguideline[Filter]) AND (2016:2022[pdat]))
Search: **child adolescent dietary guidelines dairy** Filters: **Guideline, Practice Guideline, from 2016 - 2024**

(("child"[MeSH Terms] OR "child"[All Fields] OR "children"[All Fields] OR "child s"[All Fields] OR "children s"[All Fields] OR "childrens"[All Fields] OR "childs"[All Fields]) AND ("adolescences"[All Fields] OR "adolescency"[All Fields] OR "adolescent"[MeSH Terms] OR "adolescent"[All Fields] OR "adolescence"[All Fields] OR "adolescents"[All Fields] OR "adolescent s"[All Fields]) AND ("nutrition policy"[MeSH Terms] OR ("nutrition"[All Fields] AND "policy"[All Fields]) OR "nutrition policy"[All Fields] OR ("dietary"[All Fields] AND "guidelines"[All Fields]) OR "dietary guidelines"[All Fields]) AND ("dairies"[All Fields] OR "dairy"[All Fields] OR "dairy s"[All Fields] OR "dairying"[MeSH Terms] OR "dairying"[All Fields])) AND ((guideline[Filter] OR practiceguideline[Filter]) AND (2016:2022[pdat]))
Search: **child adolescent food-based dietary guidelines** Filters: **Guideline, Practice Guideline, from 2016 - 2024**
(("child"[MeSH Terms] OR "child"[All Fields] OR "children"[All Fields] OR "child s"[All Fields] OR "children s"[All Fields] OR "childrens"[All Fields] OR "childs"[All Fields]) AND ("adolescences"[All Fields] OR "adolescency"[All Fields] OR "adolescent"[MeSH Terms] OR "adolescent"[All Fields] OR "adolescence"[All Fields] OR "adolescents"[All Fields] OR "adolescent s"[All Fields]) AND "food-based"[All Fields] AND ("nutrition policy"[MeSH Terms] OR ("nutrition"[All Fields] AND "policy"[All Fields]) OR "nutrition policy"[All Fields] OR ("dietary"[All Fields] AND "guidelines"[All Fields]) OR "dietary guidelines"[All Fields])) AND ((guideline[Filter] OR practiceguideline[Filter]) AND (2016:2022[pdat]))

Supplementary Table S3. Excluded articles and reasons.

Number	Bibliography	Reason
1	Maneschy, I., Jimeno-Martínez, A., Miguel-Berges, M. L., Rupérez, A. I., Ortega-Ramiréz, A. D., Masip, G., & Moreno, L. A. (2024). Eating Behaviours and Dietary Intake in Children and Adolescents: A Systematic Review. Current nutrition reports, 10.1007/s13668-024-00544-w. Advance online publication. https://doi.org/10.1007/s13668-024-00544-w.	Does not meet inclusion criteria.
2	Chaudhary A, Sudzina F, Mikkelsen BE. Promoting Healthy Eating among Young People-A Review of the Evidence of the Impact of School-Based Interventions. Nutrients. 2020 Sep 22;12(9):2894. doi: 10.3390/nu12092894. PMID: 32971883; PMCID: PMC7551272.	Interventions
3	Lassi ZS, Moin A, Das JK, Salam RA, Bhutta ZA. Systematic review on evidence-based adolescent nutrition interventions. Ann N Y Acad Sci. 2017 Apr;1393(1):34-50. doi: 10.1111/nyas.13335. PMID: 28436101.	Interventions
4	Linda Van H, Jo Ann SC, Lawrence JA, Lora EB, Christina E, Wahida K, et al. Recommended Dietary Pattern to Achieve Adherence to the American Heart Association/American College of Cardiology (AHA/ACC) Guidelines: A Scientific Statement From the American Heart Association. Circulation. 2016;134(22):e505-e29. doi: doi:10.1161/CIR.0000000000000462 %U https://www.ahajournals.org/doi/abs/10.1161/CIR.0000000000000462.	Does not meet inclusion criteria.
5	Guidelines for drinking-water quality: fourth edition incorporating the first and second addenda. Geneva: World Health Organization; 2022. Licence: CC BY-NC-SA 3.0 IGO.	It does not include required information. Type of study.
6	Control y seguimiento de la nutrición, el crecimiento y desarrollo de la niña y del niño menor de 5 años. México: Instituto Mexicano del Seguro Social; 2 de diciembre de 2015	Publication prior to that established in the protocol.

Supplementary Table S4. Quality appraisal of included studies.

First author	Year	Included and excluded studies list	Characteristics of the studies	Quality appraisal	Report of conflict of interest	Funding report	Score (AMSTAR, EAP, RIGHT)	Overall quality
Lisa Te Morenga	**2017**	Yes	Yes	Yes	Yes	Yes	Critically low	Low
Carmela de Llamas	**2019**	Yes	Yes	Yes	Yes	Yes	Critically low	Low
Magdalini Patseadou	**2020**	Yes	Yes	Yes	Yes	Yes	Critically low	Low
Carol Boushey	**2020**	Yes	Yes	Yes	Yes	Yes	Critically low	Low
Taylor C. Wallace	**2021**	Yes	Yes	Yes	Yes	Yes	High	High
Beatriz Teixeira	**2022**	Yes	Yes	Yes	No	No	High	High
Natasa Fidler Mis	**2017**	No	No	No	Not clear	Not clear	Positive	High
Katarzyna Dereń	**2019**	No	No	No	Not clear	Not clear	Positive	High
National Health and Nutrition Organizations	**2021**	No	No	No	Not clear	Not clear	Positive	High
Deanna M. Hoelscher	**2022**	No	No	No	No	Not clear	Positive	High
Instituto Mexicano del Seguro Social	**2016**	No	No	No	No	No	Unclear	Unclear
World Health Organization	**2017**	No	No	No	No	No	Unclear	Unclear
U.S. Department of Agriculture and U.S. Department of Health and Human Services	**2020**	No	No	No	No	No	Unclear	Unclear
World Health Organization	**2022**	No	No	No	No	No	Unclear	Unclear
